# Dynamic surveillance of tamoxifen‐resistance in ER‐positive breast cancer by CAIX‐targeted ultrasound imaging

**DOI:** 10.1002/cam4.2878

**Published:** 2020-02-12

**Authors:** Ying Li, Xiaoyu Chen, ZhiWei Zhou, Qing Li, Kenneth D. Westover, Meng Wang, Junjun Liu, Sheng Zhang, Jin Zhang, Bo Xu, Xi Wei

**Affiliations:** ^1^ Breast Cancer Center Key Laboratory of Cancer Prevention and Therapy Tianjin Medical University Cancer Institute and Hospital National Clinical Research Center for Cancer Tianjin's Clinical Research Center for Cancer Tianjin China; ^2^ Department of Diagnostic and Therapeutic Ultrasonography Key Laboratory of Cancer Prevention and Therapy Tianjin Medical University Cancer Institute and Hospital National Clinical Research Center for Cancer Tianjin's Clinical Research Center for Cancer Tianjin China; ^3^ Department of Radiation Oncology and Biochemistry University of Texas Southwestern Medical Center at Dallas Dallas TX USA; ^4^ Cancer Center Daping Hospital and Research Institute of Surgery Third Military Medical University Chongqing China

**Keywords:** breast cancer, hypoxia, PLGA‐PEG‐mAb_CAIX_ nanobubbles, tamoxifen resistance, ultrasonographic imaging

## Abstract

Tamoxifen‐based hormone therapy is central for the treatment of estrogen receptor positive (ER^+^) breast cancer. However, the acquired tamoxifen resistance, typically co‐exists with hypoxia, remains a major challenge. We aimed to develop a non‐invasive, targeted ultrasound imaging approach to dynamically monitory of tamoxifen resistance. After we assessed acquired tamoxifen resistance in 235 breast cancer patients and a list of breast cancer cell lines, we developed poly(lactic‐co‐glycolic acid)‐poly(ethylene glycol)‐carbonic anhydrase IX mono antibody nanobubbles (PLGA‐PEG‐mAb_CAIX_ NBs) to detect hypoxic breast cancer cells upon exposure of tamoxifen in nude mice. We demonstrate that carbonic anhydrase IX (CAIX) expression is associated with breast cancer local recurrence and tamoxifen resistance both in clinical and cellular models. We find that CAIX overexpression increases tamoxifen tolerance in MCF‐7 cells and predicts early tamoxifen resistance along with an oscillating pattern in intracellular ATP level in vitro. PLGA‐PEG‐mAb_CAIX_ NBs are able to dynamically detect tamoxifen‐induced hypoxia and tamoxifen resistance in vivo. CAIX‐conjugated NBs with noninvasive ultrasound imaging is powerful for dynamically monitoring hypoxic microenvironment in ER^+^ breast cancer with tamoxifen resistance.

## Graphical abstract

Targeted hypoxia ultrasonographic imaging presents an approach to dynamic surveillance of acquired antiestrogen therapeutic resistance in ER^+^ breast cancer. We established PLGA‐PEG‐mAbCAIX nanobubbles and evaluated its conjunction with ultrasonographic imaging to accomplish these goals. The early tamoxifen resistance could be dynamically monitored by the noninvasive ultrasound NBs in patients, allowing for early intervention of tamoxifen resistance.

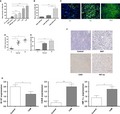

## 1 INTRODUCTION

The acquired drug resistance presents a major challenge for molecular targeted cancer therapies including endocrine therapy for breast cancer.^1^ Endocrine therapy is critical to the success of controlling hormone positive (HR^+^) breast cancers including tumors bearing the estrogen receptor (ER^+^) and/or progesterone receptor (PR^+^), both for early‐stage and metastatic breast disease.^2^, ^3^, ^4^ Tamoxifen, a selective ER mediator, is currently a standard treatment for patients with ER^+^ breast cancer.^5^ However, approximately 30% of breast cancer patients develop acquired drug resistance to tamoxifen.^6^ If hormone resistance can be dynamically monitored, it is likely that interventions are applied sooner which can translate into better patient outcomes.

Tumor microenvironment influences the behavior of tumor cells including characteristics of aggressiveness, such as invasiveness, metastasis, angiogenesis, and drug resistance.^7^ Hypoxia, a condition commonly occurred in solid tumors, is a major driver of invasiveness and metastases in breast cancer, and it is associated with resistance to chemotherapy and radiotherapy.^8^, ^9^ Cancer theranostic strategies targeting hypoxia have been explored.^10^, ^11^ In particular, imaging‐based therapies have shown promise but have not yet met all criteria required for clinical adoption. For example, dynamic contrast enhanced MRI (DCE‐MRI) and blood oxygenation level dependent (BOLD) imaging have been used to map tumor hypoxia non‐invasively in preclinical models.^12^ However, due to an indirect measurement of hypoxia or oxygen delivery, both DCE‐MRI and BOLD techniques were limited to detect hypoxia directly in solid tumors.^13^ Direct, sensitive, cost‐effective, and noninvasive imaging approaches to identifying tumor hypoxia would be useful for monitoring the development of drug resistance.

Tumor hypoxia upregulates multiple proteins including hypoxia inducible factor 1 α (HIF‐1α), carbonic anhydrase IX (CAIX), glucose transporter (GLUT1), and vascular endothelial growth factor (VEGF), resulting in cellular signal transduction that promotes adaptive changes in the microenvironment, ultimately leading to cancer cell growth and proliferation.^14^ CAIX, a downstream target protein of HIF‐1α, is overexpressed on the surface of many types of cancer cells under hypoxic conditions and has a long half‐life of several days.^15^ Given these characteristics, CAIX has been characterized as a molecular marker of hypoxia.^15^, ^16^ We explored that CAIX could be used in conjunction with targeted ultrasonographic imaging to identifying regions of tumor hypoxia in a non‐invasive manner.

With the utilization of nano‐biomaterials, ploy(lactic‐co‐glycolic acid), PLGA has been approved as a preferential candidate in the applications of non‐cytotoxic perfluorocarbon gas‐filled nano‐bubbles.^17^, ^18^ We report here the development and characterization of hypoxia‐targeted NBs loaded with a CAIX antibody (PLGA‐PEG‐mAb_CAIX_). We analyzed these particles for the ability to detect and to predict tamoxifen resistance in in vitro and in vivo cancer models. Our results provide a rational approach to monitoring hypoxia as an early biomarker in breast cancer with acquired tamoxifen resistance.

## 2 MATERIALS AND METHODS

### 2.1 Chemicals and reagents

Tamoxifen was purchased from Sigma‐Aldrich. Primary antibodies were as follows: CAIX (1:1000 dilution, ab108351, Abcam), HIF‐1α (1:500 dilution, ab51608, Abcam), VEGF (1:500 dilution, ab46154, Abcam), GLUT1 (1:500 dilution, ab115730, Abcam), ERα (1:1000 dilution, sc‐542), and Ki‐67 (1:500 dilution, REK0128, Ruierkang Company). The poly(ethylene glycol) (PEG) and PLGA were obtained from Rebone and Daigang Biomaterials Company (Shanghai).

### 2.2 Cell lines

MDA‐MB‐231, MCF‐7, and T47D cell lines were obtained from American Type Culture Collection (ATCC). Cells were cultured in MEM supplemented with 10% fetal bovine serum (FBS, Thermo Scientific) at 37°C with 5% CO_2_. Cells were grown as monolayers and passaged twice a week. Cells were exposed to tamoxifen at concentrations of 1 and 10 µmol/L. Tamoxifen was dissolved with DMSO, and the stock concentration was 50mM. It was diluted to the effect concentration with the growth medium.The control groups also have same concentration DMSO (0.02% or 2% in the in vitro or in vivo experiment) as the experimental groups.

### 2.3 Synthesis of PLGA‐B‐PEG‐COOH

The 0.4 mmol of N, N'‐Dicyclohexylcarbodiimide (DCC) (Juner (Zibo) International Trade Co.) was dissolved in 8 mL of diethylene oxide (THF) (Renown Chemicals Co,. Ltd) and then was added into a solution containing 0.1 mmol of PLGA (molecular weight: 10.0 kDa, LA: GA 50:50, Jinan Daigang Biomaterial Co.). 0.3 mmol of N‐Hydroxysuccinimide (NHS) (Sigma‐Aldrich Chemical Co.) in cooled THF (2 mL) with a drop of triethylamine (TEA, 40 μL) (Sigma‐Aldrich Chemical Co.) was added into PLGA solution at 0°C. The solution was then warmed to room temperature under N_2_. After 2 days, the mixture was poured into excessive cold ethanol (150 mL) under vigorous stirring. The precipitate was filtered out and washed with cold ethanol three times. The filtrate was regarded as the production PLGA‐NHS. The PLGA‐NHS (0.1 mmol) and NH_2_‐PEG‐COOH (0.12 mmol) (molecular weight: 5.4 kDa, Rebone Biomaterials Co.) were dissolved in cooled THF at 0°C and then were warmed to room temperature for 24 hours. The products were precipitated in anhydrous ethanol, centrifuged and dried in vacuum. The final production was considered as PLGA‐b‐PEG‐COOH.

### 2.4 Preparation of targeted nanobubbles

Nanobubbles (NBs) were prepared from an amphiphilic biomaterial, with PLGA‐b‐PEG‐COOH polymer by double emulsion (water/oil/water) evaporation. First, PLGA‐b‐PEG‐COOH (900 mg) was dissolved in dichloromethane (9 mL) at a final concentration of 100 mg/mL. The solution was poured into deionized water (1 mL) and stirred at 10 000 rpm for 5 minutes to form an emulsion. The emulsion was added into 50 mL poloxamer aqueous solution (0.35%), and stirred at 18 000 rpm for 5 minutes to form a second emulsion. After stirred over night to remove dichloromethane, the mixture was transferred into a dialysis membrane (100 kDa cut‐off), and dialyzed against 5000 mL of double distilled water for 36 hours at 4°C. Then, the solution was transferred into a 100 mL flask. The 1‐Ethyl‐3‐(3‐dimethyllaminopropyl) carbodiimide hydrochloride (EDC 0.4 mmol) (from Sigma‐Aldrich Chemical Co.) and NHS (0.4 mmol) were added into the solution at 4°C. The solution was allowed to warm to room temperature and stirred for 24 hours. The mixture was then filtered by microfiltration membrane (0.2 μm cut‐off) and was washed by deionized water three times. The precipitate was dissolved into phosphate‐buffered saline (PBS, 0.01 mol/L, pH 7.4) and incubated with 0.2 mmol of biotin‐NH_2_ (Sigma‐Aldrich Chemical Co.) for 2 hours. The mixture was then filtered by microfiltration membrane (0.2 μm cut‐off) and was washed by deionized water three times. The sample was added into glycerine and freeze‐dried to seal in a vial filled with perfluorocarbon gas to store at 4°C. The morphology and size of NBs were evaluated by transmission electron microscopy as previously described.^19^

1 mL of nanobubbles solution (1 mg/mL) was incubated with streptavidin for 24 hours at 4°C to obtain streptavidin‐coated NBs and the concentration is 1 × 10^9^/mL. The biotin‐fluorescein (biotin‐FITC) (molecular weight: 732.80 and formula: C_37_H_40_N_4_O_10_S, Thermo Fisher Scientific Inc USA) was dissolved in DMSO at a final concentration of 1mg/mL. The volume of reaction solution was 1 mL The structure ‐COOH of biotin‐FITC, with four biotin‐binding sites, was activated with EDC (0.004 mmol) in order to couple with the structure of ‐NH_2_ on the specific antibody. The CAIX antibody (0.005 mmol) (mAbCAIX, Abcam, USA) was mixed into solution with biotin‐FITC (5:1) at 4°C for 8 hours in the dark room.0.01 mL of NH_4_Cl (5 mol/L) was added into the mixture dilution at 4°C for 2 hours in the dark room. The mixture was transferred into a dialysis membrane (100 kDa cut‐off), and dialyzed against 5000 mL of double distilled water for 36 hours at 4°C in the dark room for four times. The 1 mL of the streptavidin‐coated NBs were then conjugated to mAbCAIX‐FITC‐biotin (0.01 μmol) in a 1:10 ratio by overnight incubation at room temperature in dark room. Confocal fluorescence microscope (Leica, Wetzlar, Germany) was performed to observe localization of PLGA‐PEG‐mAbCAIX NBs binding with MCF‐7 cells.

### 2.5 Patient selection

We retrospectively collected tumor specimen from 235 breast cancer patients in this study who were from Tianjin Medical University Cancer Institute and Hospital (Tianjin, China) between March 2001 and April 2014. Patients were pathologically diagnosed as invasive ductal breast carcinoma. All patients enrolled developed tamoxifen‐resistant relapses at a median of 48 months after initial tumor resection. Tamoxifen resistance was defined as patients with breast cancer undergoing relapse after treatment by post‐operative tamoxifen over 2 years. Immunohistochemistry (IHC) staining of the original tumor resection tissue samples was performed as described previously.^20^ The protocol was approved by Tianjin Medical University Cancer Institute and the Hospital ethics board. Informed consent was obtained from all enrolled patients.

### 2.6 Intracellular ATP level assay

Intracellular ATP levels were examined using Luminescent ATP Detection Assay Kit (Sigma‐Aldrich) following the manufacturer's instruction. Briefly, cells were seeded into 6‐well plates at a density of 5 × 10^5^ cells/well and treated with 1 µmol/L tamoxifen or left untreated in low‐glucose and low‐serum medium for 4 hours. The treated cells were then harvested and resuspended in 1 mL of phosphate‐buffered saline. An aliquot of 50 μL of the cell suspension was mixed with 100 μL of ATP‐releasing reagent and 50 μL of distilled water in each well of a 96‐well plate. The samples (100 μL) in each well were then transferred to a white opaque 96‐well plate whose wells were pre‐filled with 100 μL of ATP assay mix. After tamoxifen treatment, cells were lysed and incubated with ATP substrate solution. Luminesce of the resulting sample was measured using a FLUOstar Omega luminometer (BMG LABTECH, the Microplate Reader Company).

### 2.7 Immunofluorescence staining in vitro

MCF‐7 cells were incubated with CAIX primary antibody at a dilution of 1:200 overnight at 4°C then mixed with fluorochrome‐conjugated secondary antibody (1:100 dilution, cell signaling) for 1 hour at room temperature in the dark. As a control, cell nuclei were stained with DAPI. Positive cells were quantitated using LSM780NLO two‐photon microscopes (Carl Zeiss).

### 2.8 RNA isolation and quantitative PCR

Total RNA was extracted from T47D and MCF‐7 cells and the cDNA was obtained using a RT reagent Kit following manufacturer's instruction (PrimeScript^TM^, Takara Bio Inc Otsu). The fluorescent real‐time PCR reaction was performed in a thermal cycler (Fast Real‐Time PCR System, Eppendorf, Co.) for 30 cycles (denaturation at 95°C for 15 seconds and annealing at 55°C for 30 seconds) in SYBR green real‐time PCT analysis by using the following primers: *HIF‐1*α (Forward: 5′‐ AACAGTGACAAAAGACCGTA and Reverse: 5′‐ ATGACTCCTTTTCCTGCTCT), *GLUT‐1* (Forward: 5′‐ GATGCGGGAGAAGAAGGTCA and Reverse: 5′‐ AGACAGCGTTGATGCCAGAC), *CAIX* (Forward: 5′‐ ACCAGACAGTGATGCTGAGTGCT and Reverse: 5′‐ CCAAAAACCAGGGCTAGGATG), and *GAPDH* (Forward: 5′‐ CCTCTGACTTCAACAGCGACAC and Reverse: 5′‐ TGGTCCAGGGGTCTTACTCC) and *CAIXsiRNA* (Forward: 5′‐GGAAGAAAUCGCUGAGGAATT and Reverse: 5′‐UUCCUCAGCGAUUUCUUCCTT). Fluorescent readings from real‐time PCR reaction products were analyzed by quantitating the difference in cycle number of crossing point (CP) between the target gene (HIF‐1α, GLUT‐1, and CAIX) and GAPDH. The changes in hypoxia markers (HIF‐1α, GLUT‐1 and CAIX) mRNA expression in tamoxifen‐treated cells were obtained by comparison with HIF‐1α, GLUT‐1, and CAIX mRNA expression in untreated cells.

### 2.9 Immunohistochemistry (IHC) analysis

IHC staining assay was performed as follows. Formalin‐fixed tissue samples were embedded in paraffin and 5 μm sections were cut. For immunohistochemistry staining, in brief, the tissue sections on coated slides were dewaxed and subjugated to antigen retrieval by boiling in 10 mmol/L sodium citrate (pH 6.0) at 130°C for 3 minutes, then pretreated with a 3% solution of hydrogen peroxide for 30 minutes, rinsed, and incubated with 5% normal goat serum for 20 minutes as a blocking agent. The sections were incubated with primary antibodies at 4°C overnight. The next day, slides were washed in PBS and incubated with the secondary antibody for 30 minutes at room temperature. All steps were preceded by rinsing of sections with PBS (pH 7.6). The chromogen was 3,3‐diaminobenzidine (DAB). The immunoreactivity of primary antibodies in tumor tissues was scored by the H‐score method.The following primary antibodies against CAIX(1:100), Ki‐67(1:100), HIF‐1α(1:100), GLUT1(1:100), and VEGF(1:100) were used for patient specimen and mice xenograft tumors staining.

### 2.10 Western blotting analysis

Cells and tissues were lysed in RIPA solution with protease inhibitor PMSF (1 mmol/L) for 30 minutes on ice. Cell or tissue lysates were centrifuged at 12 000g for 15 minutes at 4°C, and the supernatants were collected. Protein concentrations were quantified using the BCA Protein Assay according to the manufacturer's instructions. Equal amounts (20 μg) of total protein were separated by SDS‐PAGE gel (10%‐12%) at 70 V for 0.5 hour, 120 V for 1 hour and transferred to a 0.45 μm PVDF membrane at 300 mA for 60‐150 minutes. After blocking with 5% non‐fat milk in TBST buffer for 1 hour at room temperature, the membranes were incubated with primary antibody at 4°C overnight. The membranes were washed three times with TBST buffer and then incubated with peroxidase (HRP)‐conjugated secondary antibody for 1 hour at room temperature. Specific antibody binding was detected by the Chemiluminescence Kit (Millipore). Fluorescent signals were detected by a luminescent image analyzer (C‐Digit, Gene Company Limited).

### 2.11 ER positive breast cancer xenograft mice model

The 3‐4 weeks old BALB/c nude mice (15‐20 g, female, obtained from Tianjin Medical University) were implanted with MCF‐7 cell suspension with ~10^6^/cells in the left and right anterior axilla. The xenograft tumor size was calculated using the formula: volume = long diameter × short diameter^2^/2. At a tumor volume of 200 mm^3^, mice were treated with vehicle control (n = 6, 1% DMSO + saline) or tamoxifen in DMSO (n = 6, 2 mg/mL/day) via intraperitoneal injection for 7‐28 consecutive days. There were four mice groups (n = 6 for each group) with tamoxifen treatment at four different time points (7, 14, 21 and 28 days). At the end of each time point, the mice were anesthetized with 0.5% pentobarbital sodium through intraperitoneal route. After sacrificed, xenograft tumors were then removed and fixed in formalin for paraffin‐embedded sections (Epon 812, Haide Biotech Company). The embedded samples were sliced into 3‐5μm sections by ultramicrotome. In separate experiments, the nude mice were separated into different groups following different protocols. After 14‐day implantation (the mean diameter was 6.4 ± 0.7 mm; the average tumor size was 139.7 ± 5.2 mm^3^), PLGA‐PEG‐mAbCAIX NBs were intravenously injected (1 × 10^8^ NBs per mouse in a 100 μL dose consisting of 50 μL of NBs and 50 μL of saline) via tail vein after the treatment. The experimental protocol was approved by the Animal Ethics Committee of Tianjin Medical University.

### 2.12 Ultrasound imaging in vivo

Ultrasound targeted imaging was performed in vivo on day 0 for baseline scanning and then was used for different time points according to different protocols in our study after 3‐day treatment. The imaging was repeated three times a day (1, 6, and 12 hours). After mice were anesthetized, ultrasound imaging was performed using an iU22 scanner (Phillip Medical Systems) with an L12‐5 high‐frequency linear transducer for grayscale imaging and an L9‐3 transducer for contrast ultrasound imaging. Contrast dual‐image model settings were optimized as follows: mechanical index was 0.06 and the frame rate was 11 Hz. The ultrasound probe was placed at the center of the tumor at the largest transverse cross section. Tumor volumes were calculated by the formula (*V* = *ab* ^2^/2)^21^ at three probe planes. After targeted contrast agents (diluted in 100 μL saline) was intravenously injected through mice tail vein. The contrast ultrasound imaging was performed to observe the contrast echoes from targeted NBs in 30 seconds after the injection. The gap of 60 minutes was paused to allow targeted NBs to bind with hypoxic cells at targeted sites in vivo. The dynamic imaging was recorded as the DICOM format and saved on the hard disk of the scanner for post‐imaging review. The regions of interests (ROIs) covered on tumors and were analyzed by the QLAB software (Phillip Medical Systems). The intensity, the size of perfusion areas, and other parameters (arrival time, time to peak, and area under the curve) of the time‐intensity curve (TIC) based on NBs signals were also uploaded to QLAB for analysis. The average intensity of NBs was calculated and repeated three times at each point over the entire protocol. All animal experiments were approved by the Animal Care Committee of Tianjin Medical University.

### 2.13 Statistical analysis

Data are expressed as mean ± standard error. Statistical analyses were performed using SPSS (IBM SPSS Statistics 19). The Kaplan–Meier curves for PFS were performed based on the progressive survival time by median survival time. Statistical significance was calculated using the Pearson correlation test, *χ* ^2^ test, Fisher's exact test, Analysis of Variance and student's *t* test. Two‐sided *P* values < .05 were considered as statistically different.

## 3 RESULTS

### 3.1 CAIX correlates with local recurrence in breast cancer patients receiving tamoxifen

**Figure 1:**
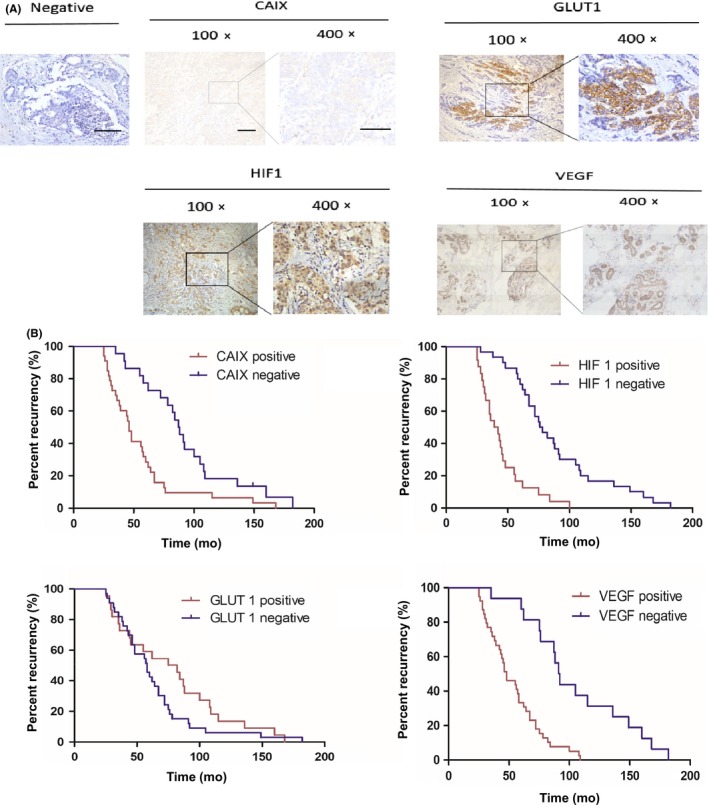
Correlation of CAIX, HIF‐1α, GLUT1, and VEGF expression with breast cancer recurrence. A, Human breast tumors samples were stained with CAIX, GLUT1, HIF‐1α and VEGF antibodies using immunohistochemistry. Magnification × 100, Scale bars: 100 µm. B, Kaplan‐Meier plot showed a correlation between HIF‐1α, CAIX, GLUT1, and VEGF and breast cancer recurrence for patients treated with tamoxifen

To evaluate the association between molecular markers of hypoxia and the development of tamoxifen resistant breast cancer, we firstly examined tissue specimens from fifty‐five (55 of 235) breast cancer patients who underwent tumor resection followed by hormone therapy with tamoxifen, in order to test hypoxic markers expression (Table 1). We evaluated HIF‐1α, CAIX, GLUT1, and the angiogenic growth factor VEGF. The expression profiles of HIF‐1α, CAIX, and VEGF were associated with the recurrence rate (Log‐rank test: *c* ^2^ = 26.50, *P < *.0001; *c* ^2^ = 10.79, *P = *.0010; *c* ^2^ = 19.94, *P < *.0001) (Figure 1A,B). There was no significant difference between GLUT1 expression and the recurrence rate (Log‐rank test: *c* ^2^ = 1.824, *P* = .1769). Due to CAIX being as cell surface marker which is able to be detected by ultrasound bubbles, we selected CAIX as the candidate for the further study. We then compared CAIX expression in 100 (100 of 235) breast cancer cases with tamoxifen resistance with 80 (80 of 235) breast cancer cases without taxmoxifen resistance. The data showed that CAIX overexpression is associated with drug resistance (*c* ^2^ = 21.47, *P* < .0001) (Figure S1).

**Table 1:** The characteristics of patients with local recurrence of breast cancer after tamoxifen treatment

Characteristics	No
Total	55
Age (rang, median,y)	49, 34‐76
Gender	
Female	55
Male	0
Pathologic type	
Invasive ductal carcinoma	50
Carcinoma simplex	5
Hormonal statues	
ER^+^	55
PR^+^	52
Hypoxia markers	
CAIX (+/−)	19/36
HIF‐1α (+/−)	24/31
GLUT‐1 (+/−)	22/33
VEGF (+/−)	39/16

### 3.2 CAIX associated with the early resistance of tamoxifen in the treatment of breast cancer in vitro

**Figure 2:**
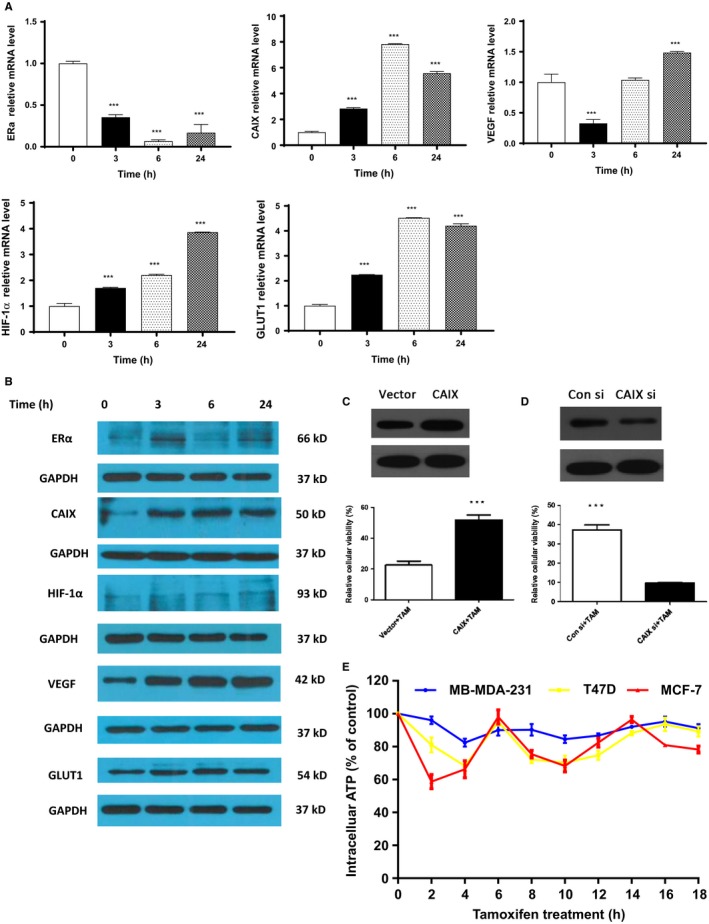
The expression of hypoxic markers upon tamoxifen exposure in vitro. The levels of mRNA (A) and protein (B) of ERα, CAIX, VEGF, GLUT1, and HIF‐1α were analyzed by ATP Bioluminescent Assay Kit, RT‐PCR, and Western blot assay, respectively. After transfection, the relative cellular viability of CAIX overexpression MCF‐7 cells (C) and CAIX siRNA MCF‐7 cells (D) exposed to tamoxifen (1 µmol/L) over 24 hours. (E) The intracellular ATP level at different time points was standardized to those of untreated cells at corresponding time points after tamoxifen (1 µmol/L) over 18 hours. Data were retrieved from three independent experiments. ***P < .001

Because CAIX is overexpressed and associated with progression‐free survival (PFS) in breast cancer patients treated with tamoxifen, we decided to test it as a potential marker for acquired tamoxifen resistance. Following the concentration optimization, the mRNA level of ERα, CAIX, HIF‐1α, GLUT1, and VEGF was tested in MCF‐7 cells after exposed to tamoxifen for 3, 6, and 24 hours (Figure 2A). The data showed that ERα mRNA level was decreased after 24‐hour tamoxifen treatment, compared to control cells (Figure 2A). However, the oscillation of ERα mRNA level was observed at 6 hours and the mRNA level rebounded at 24 hours. This finding may provide a clue to the tamoxifen resistance in vitro. Furthermore, the data showed that the increase in CAIX mRNA level was coordinated with the decrease in the ERα level, indicating that CAIX was strongly activated by hypoxia (Figure 2A). We also tested the protein level. As expected, the ERα level was fluctuated during tamoxifen treatment (0‐24 hours), which echoes the role of ER in the occurrence of tamoxifen resistance (Figure 2B). We noted that the expression of CAIX was dramatically enhanced upon the exposure to tamoxifen (Figure 2B), further supporting the notion that tamoxifen induces a hypoxic condition marker CAIX possesses the potential to predict the tamoxifen resistance under hypoxic condition. Taken together, the findings, regarding both the mRNA and protein expression results, provide evidence that CAIX, as hypoxic membrane‐associated enzyme, associated with tamoxifen tolerance in vitro.

To validate the prediction of tamoxifen resistance via CAIX expression, we upregulated CAIX expression in MCF‐7 cells after tamoxifen treatment, and found that breast cancer cells became less sensitive to tamoxifen treatment after 24 hours (Figure 2C). And vice versa, after knocking down CAIX protein in vitro, we found that MCF‐7 cells were more sensitive to tamoxifen treatment, compared to that in control siRNA group (Figure 2D). The results indicate that CAIX has the specific role in evaluation of ER^+^ breast cancer cells with sensitivity to tamoxifen.

Then, we tested the response of several breast cancer cell lines to tamoxifen exposure by measuring intracellular ATP levels. Considering that anticancer drugs inhibit glycolysis and lower intracellular ATP levels under the regulation of HIF‐1α,^22^, ^23^ we verified whether this also happens in tamoxifen treatment. ATP levels decreased in three cell lines within the first 2 hours but normalized by 6 hours before declining again in an oscillating pattern (Figure 2E). MCF‐7 cells, which expressed higher hypoxic markers, showed the most dramatic alteration with intracellular ATP levels decreasing ~50%, while T47D showed ~30% decline and MDA‐MB‐231 cells showed ~15% decrease. The oscillation in intracellular ATP level suggests that CAIX would be a sensitive marker in early tamoxifen resistance. Together, CAIX is a suitable and sensitive marker to predict early tamoxifen resistance.

### 3.3 The design, synthesis, and characterization of nano‐ultrasonographic bubbles

**Figure 3:**
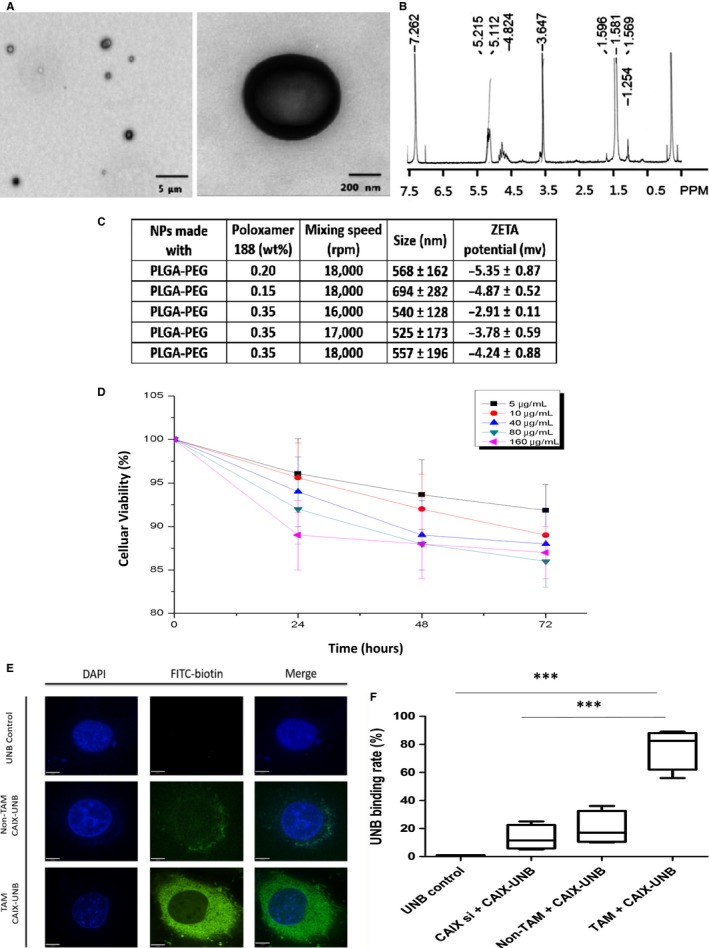
Design,synthesis,and characterization of nano‐ultrasonographic bubbles. A, The distribution and size of PLGA‐PEG NBs were demonstrated using transmission electron microscopy (scale bars: 5 µm and 200 nm). B, The PLGA‐PEG NBs were detected by nuclear magnetic resonance spectroscopy. C, The mean size and zeta potential of NBs were shown on the table. D, The MTT assay demonstrated the toxic effect of PLGA‐PEG NBs at concentrations of 5, 10, 40, 80, 160 μg/mL on MCF‐7 cells over 72 hours. E, The binding of FITC‐CAIX NBs to MCF‐7 cells in the presence or absence of tamoxifen (1 μmol/L) was shown on the graph. The fluorescence of FITC‐CAIX NBs was detected under confocal microscope. Magnification: 1500×. Scale bars: 10 µm. F, The binding rate of FITC‐CAIX NBs to MCF‐7 cells, including UNB control, CAIX siRNA MCF‐7, non‐tamoxifen and tamoxifen treatment groups, was calculated in the plot. ***P < .001. TAM: tamoxifen; UNB: ultrasonographic nanobubbles

In order to detect the dynamics of developing tamoxifen resistance via a noninvasive approach, we utilized ultrasonographic NBs. The distribution and well‐defined spherical morphology of NBs were detected via nuclear magnetic resonance spectroscopy and transmission electron microscopy (Figure 3A,B). The mean diameter of NBs was in a range from 525 ± 173 nm to 694 ± 282 nm, with zeta potential ranging from −5.35 ± 0.87 to −2.91 ± 0.11 mv (Figure 3C). The NBs was tested with a >85% viability in cellular treatment in the MTT assay, showing a good histocompatibility (Figure 3D). Moreover, NBs are able to penetrate the gaps between vessel endothelial cells via a tail vein injection in the mice bearing subcutaneous tumors described in previous study.^17^

To test the role of hypoxia‐targeted ultrasonographic NBs‐loaded with the CAIX antibody (PLGA‐PEG‐mAb_CAIX_) in detection of resistant breast cancer cells treated by tamoxifen, we first conjugated FITC‐biotin with targeted ultrasonographic NBs to be green fluorescent visible under a confocal microscope. We found that PLGA‐PEG‐mAb_CAIX_ NBs easily and specifically enriched at the surface and cytoplasm of MCF‐7 cells in response to tamoxifen treatment compared with the binding rate between NBs and CAIX knockdown cell surface (Figure 3E,F), indicating that CAIX‐targeted nano‐ultrasonographic probe PLGA‐PEG‐mAb_CAIX_ NBs have relative high specificity and enable to detect hypoxic breast cancer cells upon the exposure to tamoxifen in vitro. Collectively, the designed PLGA‐PEG‐mAb_CAIX_ NBs present a great potential for detection of early tamoxifen resistance**.**

### 3.4 Correlating ultrasonographic hypoxic imaging with early tamoxifen resistance in vivo

**Figure 4:**
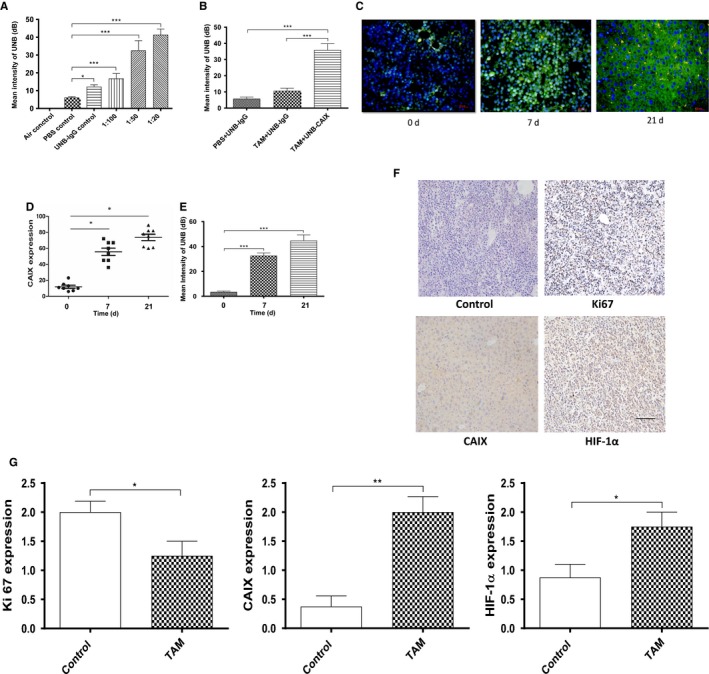
Optimization of PLGA‐PEG‐mAb_CAIX_ NBs for hypoxia detection in vivo. (A) In vivo ultrasound detection of intensity of UNBs in different mice groups (each group n = 4) treated by tamoxifen (20 mg/kg), including air control, PBS control, UNB‐IgG control; and UNB‐CAIX at different dilutions (1:100, 1:50, and 1:20). The mean intensity of NBs was measured within xenograft tumors under local hypoxic condition by ultrasonographic imaging. (B) In vivo ultrasound detection of intensity of UNBs in different groups (each group n = 6), including PBS + UNB‐IgG, TAM + UNB‐IgG, and TAM + UNB‐CAIX. The mean intensity was measured within xenograft tumors under tamoxifen treatment (20 mg/kg) and CAIX‐NBs showed the highest intensity in xenograft tumors, compared to control groups by ultrasound. (C&D) Comparison of CAIX expression and intensity of UNB‐CAIX in mice bearing MCF‐7 xenograft tumors. After tumor local vascular blockage, frozen sections were prepared and analyzed for expression of CAIX. Representative images (with fluorescein) showed the nuclear staining (blue) and the membranous staining of CAIX (green). Magnification: 200×. Scale bar: 25 µm. (E) The intensity of NBs was analyzed within tumors due to increased hypoxia induced by tumor local vascular blockage (0, 7, and 21 days). (F) IHC staining was presented for Ki67, CAIX, and HIF‐1α. Magnification: 200X; Scale bar: 200µm. (G) Bar graphs showed the expression of Ki67, CAIX, and HIF‐1α in tamoxifen‐treated and control tissue samples. *P < .05; **P < .01; and ***P < .001. TAM: tamoxifen; UNB: ultrasonographic nanobubbles

We then investigated hypoxia‐targeted nano‐ultrasonographic PLGA‐PEG‐mAb_CAIX_ NBs in the assessment of early resistance of tamoxifen treatment in nude mice bearing MCF‐7 tumors. In comparison to the mean intensity of control NBs, the optimal concentration of CAIX‐NBs was 1:20; and CAIX‐loaded NBs exhibited substantial advantage in intensity under ultrasound examination, compared to IgG‐loaded NBs in the presence or absence of tamoxifen (Figure 4A,B). The xenograft tumor staining showed that CAIX expression was significantly increased within tumors after 7‐day tamoxifen treatment (20 mg/kg) (Figure 4C), which was in agreement with the mean intensity of CAIX‐NBs by ultrasound imaging (Figure 4D,E) under hypoxic environment induced by tumor local vascular blockage. Furthermore, Ki67, CAIX, and HIF‐1α stained by IHC in tumors showed that the Ki67 level was decreased, whereas CAIX and HIF‐1α were both increased after hypoxia (Figure 4F,G).

**Figure 5:**
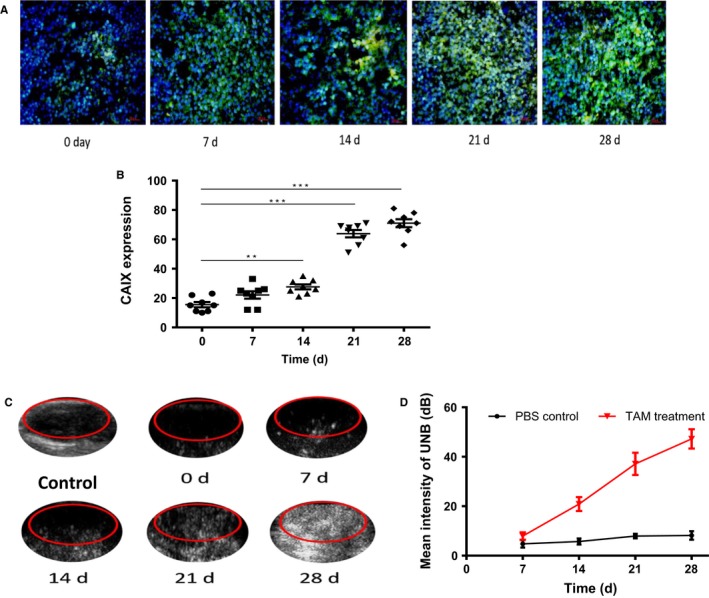
Detection of tamoxifen‐induced hypoxia using PLGA‐PEG‐mAb_CAIX_ NBs. The plot showed the (A) The green fluorescence graphs indicated CAIX expression upon the exposure to tamoxifen (20 mg/kg). Magnification: 200×. Scale bar: 25 µm. (B) Bar graphs showed the expression level of CAIX in tumors. (C) Representative ultrasound images of PLGA‐PEG‐mAb_CAIX_ NBs within tumors were detected by ultrasound. (D) Intensity of NBs shown in plots was analyzed within tumors upon tamoxifen treatment. **, *P* < .01 and ***, *P* < .001. TAM: tamoxifen

In comparison to the control, tamoxifen resistance led to tumor rapid growth after 21‐day treatment in vivo. Moreover, tumors bearing nude mice were refractory to the further tamoxifen treatment, evident from a stabilized tumor volume during the further treatment period. As a matter of fact, during the entire treatment period of 28 days, we observed an increase in green fluorescence as tamoxifen treatment continued (Figure 5A), in which there was a dramatic rise after 21‐day treatment (Figure 5A). This was coincident with the increase in CAIX expression upon the exposure to tamoxifen in vivo (Figure 5B). These results suggest that dynamic change in tumor hypoxic environment, marked by CAIX expression, was enhanced due to tamoxifen resistance. Ultrasound imaging showed that CAIX‐targeted ultrasonographic NBs were accumulated within tumors and the mean intensity of the NBs was increased after tamoxifen treatment, compared to the control (Figure 5C,D). The intensity of NBs was correlated with the pattern of CAIX expression. Collectively, tamoxifen resistance is along with hypoxic dynamic increase. The relationship among CAIX, hypoxia, and tamoxifen treatment provides a rationale for the application of NBs in monitoring the dynamics of early acquired tamoxifen resistance.

## 4 DISCUSSION

Hypoxic tumor microenvironment is associated with drug resistance to cancer therapies, such as chemotherapy and radiotherapy.^7^, ^9^, ^24^, ^25^ This is true for tamoxifen resistance during primary therapy of ER^+^ breast cancer.^9^ If we can monitor the dynamic development of tamoxifen resistance, we might be able to reverse/prevent the resistance and therapeutic failure. In the present study, we have developed a NBs‐loaded with the antibody against CAIX, a biomarker for hypoxic cells, to monitor the development of tamoxifen resistance both in vitro and in vivo models. Under tamoxifen treatment‐induced hypoxic condition, CAIX was specifically increased in ER^+^ cells, rendering that targeting CAIX would be a promising strategy to predict tamoxifen resistance in ER^+^ breast cancer therapy.

Tamoxifen therapy has been the mainstay of endocrine therapy for both early and advanced ER^+^ breast cancer patients.^26^ However, the effectiveness of tamoxifen treatment is limited due to drug resistance, and consequently patients are refractory to the tamoxifen treatment, which presents a significant clinical hurdle.^1^ It spurs the development of novel theranostic approaches to early diagnosing and effectively treating breast cancer. Among the predictive biomarkers, ER status appears to act as a gold standard for selecting patients with tamoxifen resistance. The ER status reverts to negatively imply occurrence of endocrine resistance.^27^ On the other hand, regions of low oxygen (hypoxia) are not only the common feature of solid tumors.^8^ but also a marker for drug resistance because of absent angiogenesis and oxygen against drug transmission. As a matter of fact, hypoxia has been implicated in cancer cell growth and metastasis and a negative prognostic and predictive factor, due to the various contributions to chemoresistance and radioresistance.^9^, ^10^

Thus, acquired tamoxifen resistance renders patients refractory to further tamoxifen treatment that can be explained by the hypoxia microenvironment induced by tamoxifen, with the interaction of multiple hypoxic factors.^28^, ^29^ Early detection of acquired tamoxifen resistance is urgent for physicians to optimize clinical strategies and achieve therapeutic outcome.^26^ Emerging evidence has shown that targeting CAIX would be a promising strategy for hypoxia imaging in cancers.^30^, ^31^, ^32^ Increasing number of studies have shown the role of CAIX in creating a favorable acidic microenvironment that facilitates tumor cell growth, invasion, and survival.^33^ As a membrane‐bound enzyme, CAIX is selected as a candidate for hypoxia microenvironment detection in the present study, based on the association between the expression profile of CAIX and patients with local recurrence after tamoxifen treatment. We successfully developed PLGA‐PEG NBs targeted CAIX for detecting hypoxia regions within tumors, with small size to penetrate microvessels and arrive at the surface of cells, which was able to detect breast cancer acquired tamoxifen resistance in vitro and in vivo. The findings have shown that CAIX mRNA and protein level was increased at the six‐hour period due to tamoxifen resistance, suggesting that CAIX expression enables to be a potential marker for early response of acquired tamoxifen resistance.

Intriguingly, in the xenograft mice model, we found that tumor started rapidly growing again after 21 days treatment with tamoxifen, indicating a dynamic development of tamoxifen resistance in the nude mice. The mean intensity of CAIX‐NBs was dramatically increased after 21‐day tamoxifen treatment. CAIX‐NBs was able to detect signals of tamoxifen‐induced hypoxia, and the results are in consistent with CAIX protein expression in tumor samples.

## 5 CONCLUSION

We successfully developed PLGA‐PEG‐mAb_CAIX_ ultrasound NBs as a targeted ultrasonographic probe to detect hypoxic environment in ER^+^ breast cancer. The early tamoxifen resistance could be dynamically monitored by the non‐invasive ultrasound NBs in patients, allowing for early intervention of tamoxifen resistance.

## Data Availability

I confirm that my article contains a Data Availability Statement even if no data is available (list of sample statements) unless my article type does not require one.
