# Supplementary figures and images for: Dynamic surveillance of tamoxifen‐resistance in ER‐positive breast cancer by CAIX‐targeted ultrasound imaging
Source: Cancer Med. 2020 Feb 12;9(7):2414–26. doi: 10.1002/cam4.2878 (PMC7131861; doi:10.1002/cam4.2878)

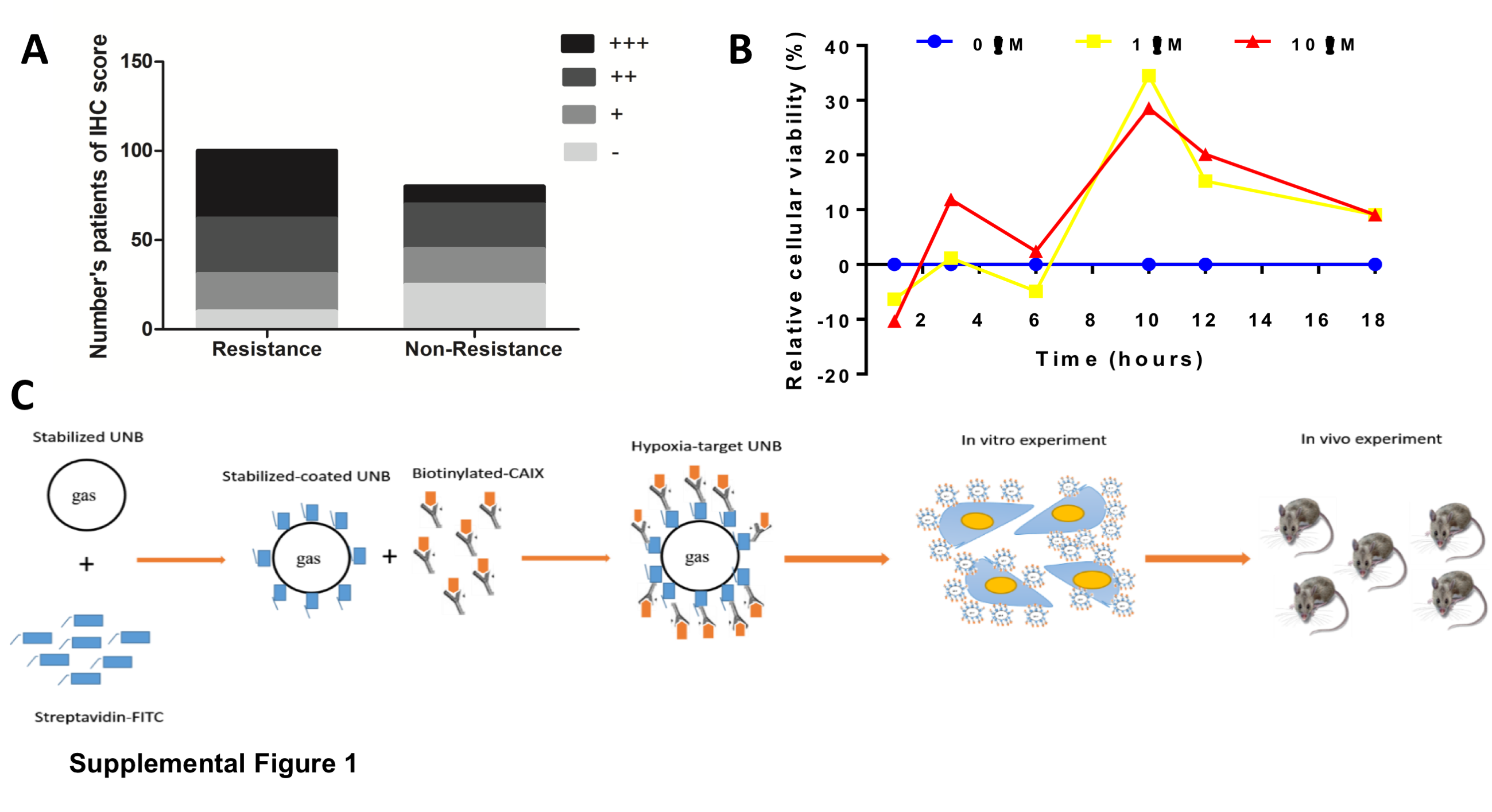

Supplement: Supplementary file 1 [file CAM4-9-2414-s001.tiff]

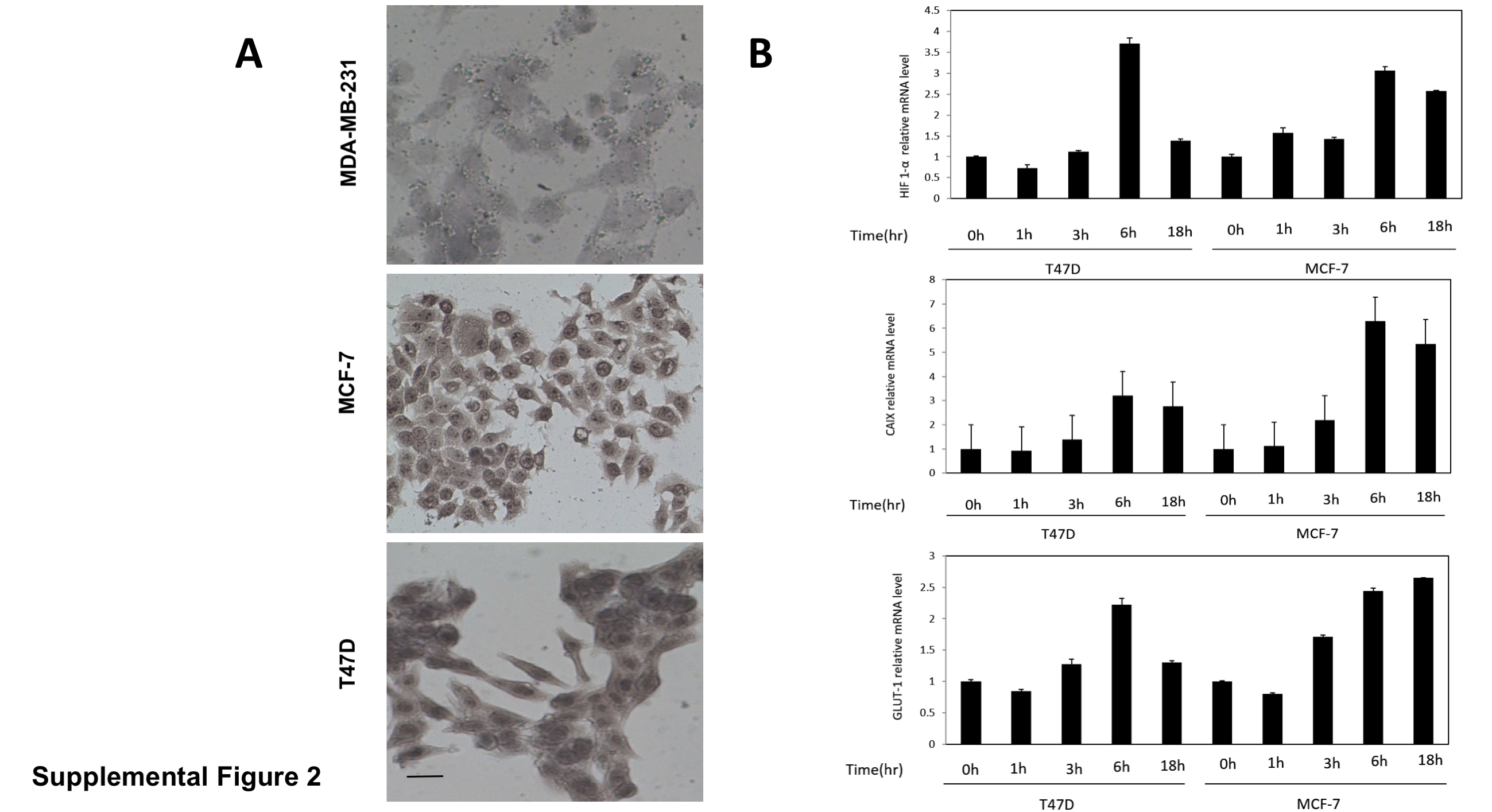

Supplement: Supplementary file 2 [file CAM4-9-2414-s002.tiff]
